# Clinical presentation and prognostic factors of *Streptococcus pneumonia*e meningitis according to the focus of infection

**DOI:** 10.1186/1471-2334-5-93

**Published:** 2005-10-27

**Authors:** Christian Østergaard, Helle Bossen Konradsen, Susanne Samuelsson

**Affiliations:** 1 National Center for Antimicrobials and Infection Control, Statens Serum Institut, Artillerivej 5, Copenhagen S, Denmark; 2 Department of Bacteriology, Mycology and Parasitology, Statens Serum Institut, Artillerivej 5, Copenhagen S, Denmark; 3 Department of Epidemiology, Statens Serum Institut, Artillerivej 5, Copenhagen S, Denmark

## Abstract

**Background:**

We conducted a nationwide study in Denmark to identify clinical features and prognostic factors in patients with *Streptococcus pneumoniae *according to the focus of infection.

**Methods:**

Based on a nationwide registration, clinical information's was prospectively collected from all reported cases of pneumococcal meningitis during a 2-year period (1999–2000). Clinical and laboratory findings at admission, clinical course and outcome of the disease including follow-up audiological examinations were collected retrospectively. The focus of infection was determined according to the clinical diagnosis made by the physicians and after review of the medical records.

**Results:**

187 consecutive cases with *S. pneumoniae *meningitis were included in the study. The most common focus was ear (30%), followed by lung (18%), sinus (8%), and other (2%). In 42% of cases a primary infection focus could not be determined. On admission, fever and an altered mental status were the most frequent findings (in 93% and 94% of cases, respectively), whereas back rigidity, headache and convulsion were found in 57%, 41% and 11% of cases, respectively. 21% of patients died during hospitalisation (adults: 27% vs. children: 2%, Fisher Exact Test, *P *< 0.001), and the causes of death were due to neurological – and systemic complications or the combination of both in 8%, 5% and 6% of cases, respectively. Other causes (e.g. gastrointestinal bleeding, incurable cancer) accounted for 2% of cases. 41% of survivors had neurological sequelae (hearing loss: 24%, focal neurological deficits: 16%, and the combination of both: 1%). The mortality varied with the focus of the infection (otogenic: 7%, sinusitic: 33%, pneumonic: 26%, other kind of focus: 50%, no primary infection focus: 21%, Log rank test: *P *= 0.0005). Prognostic factors associated with fatal outcome in univariate logistic regression analysis were advanced age, presence of an underlying disease, history of headache, presence of a lung focus, absence of an otogenic focus, having a CT-scan prior to lumbar puncture, convulsions, requirement of assisted ventilation, and alterations in various CSF parameters (WBC <500 cells/μL, high protein levels, glucose levels<1 mmol/L, low CSF/blood glucose levels), *P *< 0.05. Independent prognostic factor associated with fatal outcome in multivariate logistic regression analysis was convulsions (OR: 4.53, 95%CI: (1.74–11.8), p = 0.002), whereas presence of an otogenic focus was independently associated with a better survival (OR: 6.09, 95%CI: (1.75–21.2), *P *= 0.005).

**Conclusion:**

These results emphasize the prognostic importance of an early recognition of a predisposing focus to pneumococcal meningitis.

## Introduction

Although treatment regimens for *Streptococcus pneumoniae *meningitis are still improving (e.g. adjunctive therapy with corticosteroids [1]), the mortality rate has not changed over half a century and remains as high as ~25% with neurological sequelae in up to half of survivors [2]. Pneumococcal meningitis is secondary to a primary infection focus (e.g. ear focus in ~30%, lung focus in ~25%, sinusitic focus in ~10%), and bacteraemia is present in up to 3/4 of all cases [3-6]. Consequently, the causes of death from pneumococcal meningitis may be multifactoral and due to both neurological complications (e.g. brain herniation, seizures) and systemic complications (e.g. septic shock, multiorgan dysfunction) [5,7,8]. Therefore, predisposing condition such as the focus of the infection may be of significant importance for the outcome of the disease [5,6,9]. However, no previous studies have to our knowledge addressed clinical features of pneumococcal meningitis according to the focus of infection.

Several risk factors associated with a poor clinical outcome of pneumococcal meningitis such as advanced age, presence of an underlying disease, pneumonia or bacteraemia, decreased mental status, delay in initiation of antibiotic therapy, and alterations in various CSF parameters (e.g. low CSF glucose levels, low CSF WBC) have been identified in previous studies, but have been inconsistent findings [3-5,9-17]. However, previous studies have been relatively small in size or did not apply multivariate statistics in their risk factor analysis, and may not be comparable due to differences in study population (e.g. children vs. adults, patients admitted to intensive care units vs. all cases), which may well explain conflicting results.

The aim of the present nationwide study of 187 consecutive Danish cases with pneumococcal meningitis over a two-year period (1999–2000) was to investigate clinical features according to the focus of infection. Moreover, we wanted to clarify whether the focus is an independent prognostic factor for the outcome of pneumococcal meningitis.

## Methods

### Identification of patients

Pneumococcal meningitis is a notifiable disease in Denmark, and all cases are reported to the Department of Epidemiology, Statens Serum Institut (SSI). In addition, all pneumococcal isolates obtained from CSF and blood are sent to the National Reference Laboratory at the Streptococcus Unit, SSI for serotyping and confirmatory antibiotic susceptibility testing. If a case with a positive CSF isolate were not reported initially, a request was sent out to ensure the reporting of the case. Therefore, the study represents a nationwide collection of consecutive cases with pneumococcal meningitis, and all notified patients with onset of pneumococcal meningitis during the period 1. January 1999 to 31. December 2000 were included in the study. Pneumococcal meningitis was defined as a CSF culture with *S. pneumoniae *or CSF pleocytosis (≥ 10 leukocytes/mL) in conjunction with a blood culture of *S. pneumoniae *[18].

### Data collection

Clinical and laboratory information is provided with the notification form (i.e. diagnosis, time of onset of symptoms, clinical features, vaccination status, time of lumbar puncture and the results of CSF microscopy and CSF/blood culture), and hospital discharge records have prospectively been collected from all reported cases. Additional medical records including laboratory findings at admission, clinical course and outcome of the disease, as well as results from follow-up audiological examinations were collected retrospectively. Admission and discharge records including time of death were retrospectively controlled in the Danish civil registration database and in the national hospital registration database (Grønne system).

A total of 178 out of 188 isolates (95%) obtained from CSF and blood of the reported cases were referred to the National Reference Laboratory at the Streptococcus Unit, SSI for serotyping and confirmatory antibiotic susceptibility testing. Serotyping was performed by the Quelling reaction using type-specific pneumococcal rabbit antisera (Pneumosera^®^, SSI). Testing for penicillin susceptibility was performed with oxacillin (1 μg disk, AB Biodisk Solna, Sweden) with subsequent determination of MIC values for all isolates with reduced susceptibility using the E-test (AB Biodisk). The results of susceptibility testing performed at the local hospital laboratory were also collected from 7 isolates that were not sent to SSI.

### Definition of the focus

The focus of infection was determined according to the clinical diagnosis made by the physicians, and was confirmed respectively by review of the medical records. 1) The presence of an otogenic focus was determined by otoscopic examination, often performed by an otologist, and did not require the isolation of pneumococci from middle ear fluid. 2) The presence of a sinusitic focus was determined after examination by an otologist and confirmed by cranial radiography of the sinuses including CT-scan/MR-scan, and did not require the isolation of pneumococci from sinus fluid. 3) The presence of a pneumonic focus was determined by clinical examination together with confirmatory chest radiography, and did not require isolation of pneumococci from sputum. 4) The presence of other foci was determined by clinical signs of infection together with isolation of pneumococci in samples from these foci. 5) No primary infection focus was considered, when no primary infection foci could be detected, and when pneumococci were not isolated from other body fluids than CSF and/or blood.

### Ethics

All protocols were approved by the local scientific ethic committee and the Danish Data Protection Agency (#2002-41-2278).

### Statistical analysis

All results are given as medians and interquartile range. Fisher's exact test was used for analysis of categorical data. For analysis of continuous data, comparison between two groups was performed by use of the non-parametric Mann-Whitney test and between more than two groups by use of the non-parametric Kruskal-Wallis test. For analysis between groups according to the focus, a Bonferroni correction of 10 was used to compensate for multiple comparisons. Survival was estimated by the methods of Kaplan-Meier and compared by the Log rank test. Relative risk for progression to death was calculated using univariate and multivariate logistic regression analysis. Only variables with more than 80% of values available and with a P-value <0.2 were tested in the multivariate analysis. *P *< 0.05 was considered significant.

## Results

### Identification of patients

A total of 187 cases with pneumococcal meningitis were identified and included in the study; one case patient was excluded, because the patient had no evidence of meningitis, and the isolation of an unencapsulated pneumococcal strain from the CSF was considered a laboratory contamination (growth in 1 out of 3 cultures). A lumbal puncture was performed on all 187 cases, except one case patient, where the diagnosis was established postmortem (autopsy showed purulent meninges and the growth of pneumococci was obtained from a swap taken from the meninges). A total of 176 out of 186 cases had a positive CSF culture with pneumococci, whereas 10 cases had a positive blood culture with pneumococci and biochemical evidence of meningitis including a CSF WBC count >10 leukocytes/mL. No significant difference in routine CSF parameters was observed between cases with or without a positive CSF culture (WBC: 1799 (290–4343) vs. 4288 (85–4713); protein: 2.9 (1.5–5.8) vs. 2.6 (1.2–8.0); glucose: 0.9 (0.3–2.6) vs. 3.5 (0.7–4.2), respectively, *P *> 0.05). The annual incidence rate of pneumococcal meningitis in Denmark was 1.73 cases per 100.000 persons.

### Clinical characteristics according to the focus (see Table 1)

**Table 1:** Characteristics of 187 patients with *S. pneumoniae *meningitis according to the focus of the infection.

% or median (25/75 percentiles) and (No/total)	All cases (N = 187)	Otogenic focus (N = 57)	Sinusitic focus (N = 15)	Pneumonic focus (N = 33)	Other foci (N = 4)	No primary infection focus (N = 78)
Sex (female/male)	91/96	34/23	9/6	15/18	3/1	30/48
Age (in years)	55 (22–69)	56 (37–71)	66 (39–73)	58 (44–70)	75 (55–81)	49 (1–64)
<16 years of age	24% (45/187)	21% (12/57)	7% (1/15)	9% (3/33)	0% (0/4)	37% (29/78)
Predisposing condition*	12 % (23/185)	11% (6/57)	27% (4/15)	6% (2/33)	0% (0/4)	15% (11/76)
Underlying disease^§^	18% (33/185)	14% (8/57)	20% (3/15)	36% (12/33)	25% (1/4)	12% (9/76)
Clinical features on admission						
Fever	93% (155/166)	98% (55/56)	92% (12/13)	100% (27/27)	75% (3/4)	95% (63/66)
Headache	41% (48/116)	44% (17/39)	63% (5/8)	40% (6/15)	0% (0/3)	39% (20/51)
Back rigidity	57% (86/151)	65% (34/52)	75% (9/12)	41% (9/22)	25% (1/4)	54% (33/61)
Decreased consciousness	94% (165/176)	98% (53/54)	86% (12/14)	93% (28/30)	100% (4/4)	92% (68/74)
Convulsion (Debut before/ after admission)	31% (54/175) 11% (19/175) 17% (29/175)	23% (13/56) 5% (3/56) 18% (10/56)	33% (5/15) 20% (3/15) 13% (2/15)	41% (12/29) 10% (3/29) 31% (9/29)	50% (2/4) 0% (0/0) 50% (2/4)	31% (22/71) 14% (10/71) 17% (12/71)
Duration of symptoms	2 days (2–4) (111/187)	2 days (1–4) (53/57)	3 days (2–7) (11/15)	3 days (2–8) (12/33)	3 days (1–5) (3/4)	2 days (2–3) (37/76)
Mechanical ventilation	58% (97/168)	49% (25/51)	50% (6/12)	77% (24/31)	100% (4/4)	54% (38/70)
CT-scan before lumbar puncture	11% (21/187)	7% (4/57)	13% (2/15)	12% (4/33)	0% (0/4)	14% (11/78)
Steroid therapy	16% (26/163)	14% (7/51)	8% (2/13)	19% (5/26)	0% (0/4)	17% (12/69)
Paraclinical findings CSF WBC (cells/μL)	1842 (291–4419) (149/187)	2844 (914–4553)^## ^(50/57)	119 (41–5489) (12/15)	497 (66–2708) (24/33)	38 (1–235) (4/4)	2475 (850–4650)¤¤ (59/78)
CSF protein (g/L)	2.7 (1.4–5.8) (123/187)	3.1 (1.7–5.9) (44/57)	4.0 (1.0–9.0) (10/15)	2.8 (1.0–6.0) (18/33)	8.2 (0.5–10) (3/4)	2.3 (1.3–4.4) (48/78)
CSF glucose (mmol/L)	0.9 (0.4–2.8) (129/187)	0.9 (0.4–2.6) (45/57)	0.7 (0.1–3.0) (11/15)	0.9 (0.3–3.2) (21/33)	2.4 (0.6–3.7) (3/4)	0.9 (0.4–2.9) (49/78)
CSF/blood glucose ratio	0.1 (0.04–0.4) (93/187)	0.1 (0.05–0.3) (29/57)	0.05 (0.01–0.4) (9/15)	0.07 (0.01–0.4) (18/33)	0.5 (0.09–0.9) (2/4)	0.2 (0.04–0.5) (35/78)
Positive CSF culture	95% (176/186)	93% (53/57)	100% (15/15)	91% (29/32)	100% (4/4)	96% (75/78)
Blood WBC (10^9 ^cells/L)	17.3 (10.5–25.7) (134/187)	17.6 (10.5–24.1) (29/57)	20.0 (8.9–26.2) (12/15)	12.6 (7.9–22.9) (23/33)	10.2 (6.7–19) (3/4)	17.9 (14.1–27.7) (50/78)
Positive blood culture	67% (124/186)	74% (42/57)	60% (9/15)	72% (23/32)	100% (4/4)	60% (46/78)
Decreased penicillin susceptibility	6% (10/183)	7% (4/57)	7% (1/14)	0% (0/32)	0% (0/4)	7% (5/75)
Death during hospitalisation	21% (39/187)	7% (4/57)**	33% (5/15)	26% (12/33)	50% (2/4)	21% (16/78)
1. Neurological causes^#^	8% (16/187)^$^	3.5% (2/57)	13% (2/15)	12% (4/33)	0% (0/4)	10% (8/78)
2. Systemic causes^¤^	5% (9/187)^$^	0% (0/57)	13% (2/15)	9% (3/33)	25% (1/4)	4% (3/78)
3. Other causes^&^	2% (3/187)	0% (0/57)	0% (0/15)	9% (3/33)	0% (0/4)	0% (0/78)
Combination of 1 and 2	6%(11/187)^$^	3.5% (2/57)	7% (1/15)	6% (2/33)	25% (1/4)	7% (5/78)
Sequelae	41% (57/138)	54% (26/48)	22% (2/9)	45% (9/20)	100% (1/1)	32% (19/60)
1. Hearing loss	24% (34/138)	33% (16/48)	11% (1/9)	20% (4/20)	0% (0/1)	22% (13/60)
2. Neurologic abnormality^ˆ^	16% (22/138)	21% (10/48)	11% (1/9)	25% (5/20)	100% (1/1)	8% (5/60)
Combination of 1 and 2	1% (1/138)	0% (0/48)	0% (0/9)	0% (0/20)	0% (0/1)	2% (1/60)
Number of days hospitalised among survivors	13 (10–20)	13 (10–19)	11 (10–36)	22 (13–32)	44	13 (11–16)

* Predisposing condition was defined a previous head trauma, liquorrhoea, dura disruption etc.

§Underlying disease was defined as previous splenectomy, presence of immunodeficit, cancer, diabetes mellitus, alcoholism, or the use of immunosuppressive drugs.

# Includes brain herniation, cerebrovascular complications.

¤ Includes septic shock, multiple-organ dysfunction.

& Includes withdrawal of care due to incurable cancer (1 patient), gastrointestinal bleeding (+/- Bilroth II operation, 2 patients).

ˆNeurologic abnormality was defined as presence of aphasia, ataxia and paresis at discharge.

$ 1 patient also had gastrointestinal bleeding/Billroth II operation.

##Significant difference vs. sinusitic cases, pneumonic cases, and cases with other foci (Mann Whitney test with Bonferonis correction *P *< 0.05).

¤¤ Significant difference vs. sinusitic cases, and cases with other foci (*P *< 0.05).

**Significant difference vs. pneumonic cases (Fisher Exact test with Bonferonis correction, *P *= 0.01).

A total of 57 out of 187 patients with pneumococcal meningitis (30%) were identified as having an otogenic focus (10 patients had mastoiditis), 15 out of 187 patients had sinusitis (8%), 33 out of 187 patients had pneumonia (18%), 4 out of 187 patients had an alternative foci (2%, e.g. septic arthritis, subphrenic abscess), whereas no primary infection focus could be found in 78 out of 187 patients (42%). A total of 13 out of the 78 patients, where no primary infection focus could be determined, had no reports of an otoscopic examination. However, none of these patients had a history of earache or suppuration from the ear, and a CT-scan showed no ear focus in 4 of these patients. Six patients with an otogenic focus had an additional focus (1 patient had sinusitis and 5 patients had pneumonia; one of these patients died), whereas 3 patients with sinusitis also had pneumonia (one of these patients died). Other predisposing conditions than the focus of the infection (e.g. previous head trauma, liquorrhoae, dura disruption) were present in 12% of cases with the highest frequency among cases with a sinusitic focus (27%), among cases, where no primary infection focus was found (15%) and among cases with an otogenic focus (11%). The retrospective review of medical records showed that 8 out of 187 cases were initially misclassified in the prospective registration.

There was no significant difference in clinical and demographic characteristic between the 5 focus groups (see Table 1), except with differences in the case fatality rate between groups (see below) and in CSF WBC (Kruskal Wallis test, *P *< 0.001). CSF WBC counts were significantly higher in otogenic cases than in sinusitic cases, than in pneumonic cases, and than in cases with other foci, and were significantly higher in cases, where no primary infection focus was determined as compared to sinusitic cases and cases with other foci (see Table 1, Mann Whitney test with Bonferonies correction, *P *< 0.05). The median age of the patients was not significantly different between groups, and the focus of infection was only in part dependent on age groups (<16 vs. ≥ 16 years): otogenic focus (27% (12/45) vs. 32% (45/142), respectively, *P *= 0.58), sinusitic focus (2% (1/45) vs. 10% (14/142), respectively, *P *= 0.12), pneumonic focus (8% (3/45) vs. 20% (30/142), respectively, *P *= 0.03), other foci (0% (0/45) vs. 3% (4/142), respectively, *P *= 0.57), and no primary infection focus (64% (29/45) vs. 35% (49/142), respectively, *P *= 0.0005). Not surprisingly was headache more frequently reported in adults than in children, and adults had more frequently an underlying disease, required more frequently assisted ventilation and had a poorer outcome than children (see Table 2, *P *< 0.05).

**Table 2:** Characteristics of 187 patients with *S. pneumoniae *meningitis according to age groups

% (No/total) or Median (25/75 percentiles)	Adults (≥ 16 years) (N = 142)	Children (<16 years) (N = 45)
Sex (female/male)	71/71	20/25
Age	61 years (50–71)	12 month (7–18)
Predisposing condition*	13% (19/140)	9% (4/45)
Underlying disease^§^	23% (32/140)	2% (1/45)**
Clinical features on admission		
Fever	97% (119/123)	95% (41/43)
Headache	57% (47/82)	3% (1/34)**
Back rigidity	61% (67/110)	46% (19/41)
Decreased consciousness	96% (131/137)	87% (34/39)
Convulsion (Debut before/ after admission)	28% (37/130) 6% (8/130) 22% (29/130)	37% (17/45) 24% (11/45)** 13% (6/45)
Duration of symptoms	2 days (2–4) (83/142)	2 days (2–6) (28/45)
Mechanical ventilation	67% (86/128)	28% (11/40)**
CT-scan preceding lumbar puncture	13% (19/142)	4% (2/45)
Steroid therapy	7% (8/121)	43% (18/42)**
Paraclinical findings CSF WBC (cells/μL)	2475 (122–4659) (109/142)	1690 (775–2622) (40/45)
CSF protein (g/L)	3.7 (2.1–6.9) (91/142)	1.6 (0.8–2.2)** (32/45)
CSF glucose (mmol/L)	0.9 (0.3–2.5) (95/142)	1.4 (0.4–3.2) (34/45)
CSF/blood glucose ratio	0.09 (0.02–0.3) (72/142)	0.2 (0.07–0.6) (21/45)
Positive CSF culture	93% (131/141)	100% (45/45)
Blood WBC (10^9 ^cells/L)	15.9 (10.1–24.8) (105/142)	20.3 (12.8–29.2) (29/45)
Positive blood culture	67% (94/141)	67% (30/45)
Decreased penicillin susceptibility	4% (5/139)	11% (5/45)
Death during hospitalization	27% (38/142)	2% (1/45)**
1. Neurological causes^#^	11% (15/142)^$^	2% (1/45)
2. Systemic causes^¤^	6% (9/142)^$^	0% (0/45)
3. Other causes^&^	2% (3/142)	0% (0/45)
Combination of 1 and 2	8% (11/142)	0% (0/45)
Sequelae	52% (50/96)	17% (7/42)**
1. Hearing loss	30% (29/96)	12% (5/42)
2. Neurologic abnormality^ˆ^	22% (21/96)	2.5% (1/42)
Combination of 1 and 2	0% (0/96)	2.5% (1/42)
Number of days hospitalised among survivors	15 (11–22)	11 (10–14)**

* Predisposing condition was defined a previous head trauma, liquorrhoea, dura disruption etc.

§Underlying disease was defined as previous splenectomy, presence of immunodeficit, cancer, diabetes mellitus, alcoholism, or the use of immunosuppressive drugs.

# Includes brain herniation, cerebrovascular complications.

¤ Includes septic shock, multiple-organ dysfunction.

& Includes withdrawal of care due to incurable cancer (1 patient), gastrointestinal bleeding (+/- Bilroth II operation, 2 patients). ˆNeurologic abnormality was defined as presence of aphasia, ataxia and paresis at discharge.

$ 1 patient also had gastrointestinal bleeding/Billroth II operation.

**Significant difference vs. adult cases (Mann Whitney test or Fisher Exact Test, *P *< 0.05.

On admission, fever and an altered mental status were present in almost all cases with pneumococcal meningitis (93% and 94%, respectively), whereas back rigidity, headache, and convulsion were significantly less frequent findings (57%, 41%, and 11%, respectively, *P *< 0.0001). Twenty-one case patients (11%) had a diagnostic CT-scan before the lumbar puncture. These patients, except one patient, who received antibiotic therapy with ceftriaxone, were initially suspected of having another intracranial disease than meningitis (e.g. apoplexy), resulting in a delay in the establishment of a correct diagnosis and initiation of antibiotic therapy. Initial or empiric antibiotic therapy included penicillin and/or a 3. generation cephalosporin and was changed according to the susceptibility of the pathogen to penicillin in most cases. Adjunctive therapy with corticosteroids was given to 16% of cases with a significantly higher treatment rate among children than among adults (see Table 2, *P *< 0.001).

### Case fatality rate

**Figure 1:**
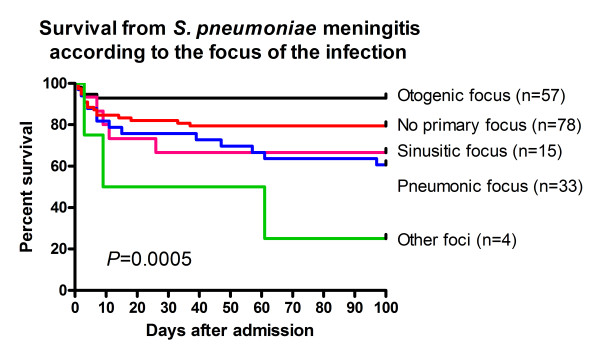
**Kaplan Meier Survival curve of 187 patients with *S. pneumoniae *meningitis in Denmark 1999–2000 according to the focus of the infection**. Otogenic focus vs. pneumonic focus, sinusitic focus, other foci, and no primary infection focus: Log rank test: *P *= 0.0002, 0.008, <0.0001, and 0.03, respectively. Other foci vs. no primary infection focus: *P *= 0.01.

**Figure 2:**
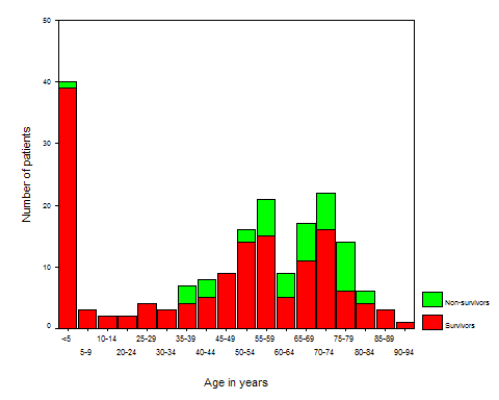
Age distribution and mortality of 187 patients with *S. pneumoniae *meningitis in Denmark 1999–2000.

**Figure 3:**
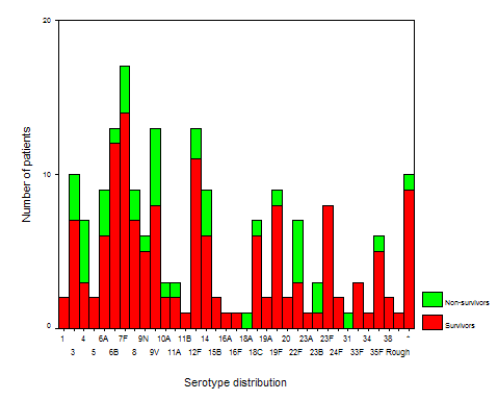
**Serotype distribution and mortality of 187 patients with *S. pneumoniae *meningitis in Denmark 1999–2000**. *10 isolates were not capsular serotyped.

The case fatality rate during the first 100 days after admission varied according to the focus of infection (Log rank test, *P *= 0.0005, see Figure 1). Patients with an otogenic focus had a significant lower case fatality rate than non-otogenic cases (within 14 days: 7% vs. 21%, Log-rank test: *P *= 0.03, within 31 days: 7% vs. 22%, *P *= 0.02, and within 100 days: 7% vs. 29%, *P *= 0.002). Adult patients had a significantly higher case fatality rate than children (see Figure 2, and Table 2, *P *= 0.0001). The case fatality rate according to serotype distribution is shown in Figure 3, and a trend was seen for different case fatality rates among serotypes. The most frequent serotypes according to the focus were as follows: Otogenic focus: serotype 8(6), 3(4), 6B(4), 19F(4), 12F(3), and 7F(3). Sinusitic focus: serotype 7F(3), 6B(2), 10A(2), and 35F(2). Pneumonic focus: serotype 4(5), 14(4), 7F(3), 8(3), and 9V(3). Other kind of focus: serotype 6A(1), 12F(1), 14(1), and 22F(1). No primary infection focus: serotype 7F(8), 6B(7), 12F(6), 23F(6), 6A(5), 9V(5), and 18C(5). Ten out of 184 isolates (5%) had reduced susceptibility for penicillin (serotype 5 (2), 6B (1), 9N (1), 9V (1), 14 (1), 15B (2), and 19F (2); no data available for 3 isolates). No significant differences between case fatality rate penicillin susceptibility were observed (*P *> 0.05).

Two out of every 3 deaths due to pneumococcal meningitis occurred within the first week after admission, and all patients died during hospitalisation, except 2 patients; 1 with severe brain damage (vegetative state) and 1 one with severe cardiovascular complications, who died 57 and 61 days after admission, respectively. These two patients were transferred to a nursing home for a short period before time of death/readmission and death at the hospital. The causes of death during hospitalisation was as follows: 41% died due to neurological causes (e.g. brain herniation, cerebrovascular complications), 23% died due to systemic causes (e.g. septic shock, multiple-organ dysfunction), 8% died due to other causes (e.g. gastrointestinal bleeding, incurable cancer) and 28% died due to a combination of systemic and neurological complications. Gastrointestinal bleeding was observed in a total of 5 patients dying from pneumococcal meningitis (2 patients had a Billroth II operation); none of these were treated with steroids. The median time to death due to neurological -, systemic -, other complications or the combination of neurological and systemic complications was 2 days (3–30), 7 days (4–9), 47 days (11–61), and 6 days (3–10), respectively (Kruskal Wallis test *P *= 0.07).

### Prognostic factors

Patients with advanced age, with an underlying disease, without an otogenic focus, with a lung focus and with convulsions, as well as patients, who had a CT-scan before lumbar puncture, and who required assisted ventilation were at increased risk for fatal outcome (within 100 days) in univariate logistic regression analysis (*P *< 0.05, see Table 3). Also, an association was found between case fatality rate and various alterations in CSF parameters (WBC< 500 cells/μL, high protein levels, glucose levels <1 mmol/L, low CSF/blood glucose ratios), *P *< 0.05. In the multivariate analysis (not including assisted ventilation therapy and variables with less than 80% available values), prognostic factors for fatal outcome due to pneumococcal meningitis were convulsions, whereas presence of an otogenic focus was independently associated with a better survival (see Table 3, *P *< 0.05). Stepwise inclusion of other possible prognostic factors did not weaken the association between a better survival and presence of an otogenic focus (data not shown) Independent prognostic factors for fatal outcome during hospitalisation were similar to the 100 days calculation (data not shown), and independent risk factors for dying within 31 days after admission were having a CT-scan preceding lumbar puncture (OR: 4.13 (1.36–12.6), *P *= 0.01), and absence of an otogenic focus (OR: 3.61 (1.09–12.0), *P *= 0.036). Not surprisingly, assisted ventilation was independently associated with fatal outcome (OR: 9.45 (3.18–28.1), *P *< 0.001) and when included in the multivariate analysis, convulsion and presence of an otogenic focus were still significant associated with a higher and lower case fatality rate at 100 days, respectively (OR: 3.15 (1.11–8.94), *P *= 0.03 and OR: 6.68 (1.71–26.1), *P *= 0.006, respectively).

**Table 3:** Prognostic clinical parameters for fatal outcome^# ^due to *S. pneumoniae *meningitis

			Univariat analysis		Multivariate	
% or median (25/75 percentiles) and (No/total)	Non-survivors N = 41	Survivors N = 146	OR^¤^	*P*-value	OR^¤^	*P*-value
Sex (female)	51% (21/41)	48% (70/146)	1.14 (0.57–2.28)	0.71		
Age (in years)	67 (56–74)	50 (1–67)	1.036 (1.02–1.06)	<0.001	1.03 (1.00–1.07)	0.05
Age ≤ 16 years	2% (1/41)	30% (44/146)	0.06 (0.008–0.44)	0.006	0.36 (0.02–7.13)	0.50
Predisposing condition*	10% (4/40)	13% (19/145)	0.74 (0.24–2.30)	0.60		
Underlying disease^§^	30% (12/40)	15% (21/145)	2.53 (1.12–5.74)	0.03	1.72 (0.58–5.08)	0.33
Admission during the first half of the year	83% (34/41)	68% (99/146)	2.30 (0.95–5.58)	0.06	1.27 (0.41–3.97)	0.68
Fever	95% (35/37)	97% (125/129)	0.56 (0.10–3.19)	0.61		
History of headache	62% (13/21)	38% (35/95)	2.79 (1.05–7.38)	0.04		
Back rigidity	55% (16/29)	57% (70/122)	0.91 (0.40–2.07)	0.84		
Decreased consciousness	98% (40/41)	93% (125/135)	3.20 (0.40–25.8)	0.46		
Convulsion	53% (19/36)	25% (35/139)	2.32 (1.07–5.04)	0.03	4.53 (1.74–11.8)	0.002
Mechanical ventilation^&^	90% (35/39)	48% (62/129)	9.45 (3.18–28.1)	<0.001		
CT-scan preceding lumbar puncture	22% (9/41)	8% (12/146)	3.14 (1.22–8.01)	0.02	2.62 (0.82–8.41)	0.11
Steroid therapy	9% (3/32)	18% (23/131)	0.49 (0.14–1.73)	0.27		
Bacteraemia	78% (31/40)	64% (93/146)	1.96 (0.87–4.43)	0.11	2.02 (0.70–5.84)	0.19
Otogenic focus	10% (4/41)	36% (53/146)	0.19 (0.06–0.56)	0.003	0.16 (0.05–0.57)	0.005
Lung focus	39% (16/41)	17% (25/146)	3.10 (1.45–6.63)	0.004	1.46 (0.53–3.97)	0.46
CSF WBC (10^9 ^cells/L)	0.32 (0.07–3.1) (33/41)	11 (0.7–4.7) (116/146)	0.88 (0.77–1.01)	0.06		
500 cells/μL	55% (18/33)	22% (26/116)	4.15 (1.84–9.36)	0.001		
CSF protein (g/L)	4.7 (2.7–8.9) (27/41)	2.4 (1.3–4.8) (96/146)	1.19 (1.07–1.33)	0.001		
CSF glucose (mmol/L)	0.4 (0.1–1.6) (29/41)	1.0 (0.4–3.1) (100/146)	0.83 (0.63–1.08)	0.17		
<1 mmol/L	76% (22/29)	52% (52/100)	3.41 (1.33–8.69)	0.01		
CSF/blood glucose ratio	0.05 (0.01–0.11) (21/41)	0.18 (0.06–0.5) (72/146)	0.01 (0.00–0.36)	0.01		
Blood WBC (10^9 ^cells/L)	14.8 (9.5–24.1) (28/41)	17.7 (10.8–26.8) (106/146)	0.98 (0.94–1.02)	0.34		

# Within 100 days after admission.

¤ OR was calculated per additional units for continuous data.

* Predisposing condition was defined a previous head trauma, liquorrhoea, dura disruption etc.

§Underlying disease was defined as previous splenectomy, presence of immunodeficit, cancer, diabetes mellitus, alcoholism, or the use of immunosuppressive drugs.

& If need for assisted ventilation was included in the multivariate analysis convulsions, absence of otogenic focus, and need for assisted ventilation were of significant importance for fatal outcome (p < 0.05, data not shown).

### Sequelae among survivors of pneumococcal meningitis

Sequelae were present in 41% (57/138) of survivors with various degree of hearing loss in ~25% of survivors, and motor sequelae such as ataxia and paresis were present in ~16% of survivors. Among survivors, adult patients had significantly more frequent sequelae than children (see Table 2, *P *< 0.001), and the median age was significantly higher among patients with – than without sequelae (57 years (50–71) vs. 29 years (1–61), respectively, *P *< 0.001). Sequelae among survivors were significantly more frequent in patients, who required assisted ventilation (65% (37/57) vs. 23% (15/65), *P *< 0.001), in patients, who were not treated with corticosteroids 47% (47/100) vs. 13% (3/23), *P *= 0.004), and patients with sequelae had significantly higher CSF protein levels (3,0 (2.1–4.6) vs. 1.6 (1.0–5.5), *P *= 0.016 and lower CSF glucose levels 0.7 (0.3–1.6) vs. 1.9 (0.6–3.4), *P *= 0.002, respectively) than in patients without sequelae.

## Discussion

The present nationwide study of 187 consecutive cases with pneumococcal meningitis in Denmark over a 2-year period (1999–2000) shows that the overall case fatality rate was 21% with a 10-fold higher mortality rate among adults than among children, which is comparable with recent reports from other industrialised countries [5,6,9,19-21]. The case fatality rate according to age groups seems to resemble previous findings obtained from patients with pneumococcal meningitis admitted to a tertiary Danish hospital during the period 1966–1976 [4] indicating that the epidemiology as well as the mortality of pneumococcal meningitis has not changed significantly over a 30–40 years period. Also, the present study confirms that predisposing conditions (e.g. dura leakage and associated foci) are found in 2 out of every 3 patients with pneumococcal meningitis [3-5]. An otogenic focus was the most frequent focus (30%) followed by a pneumonic focus (18%), a sinusitic focus (8%), whereas no primary infection focus was found in 42% of cases with pneumococcal meningitis. A high proportion of cases were children, when no primary infection focus was detected, despite of thoroughly examination for other infection foci, which could indicate that nasopharyngeal colonisation with pneumococci may be a risk factor for developing pneumococcal meningitis particular in children.

The case fatality rate varied according to the focus of infection with a significantly lower case fatality rate among otogenic cases (7%) as compared to non-otogenic cases of pneumococcal meningitis (27%). Others have reported similar trends for a better outcome of otogenic pneumococcal meningitis [4]. A lung focus was associated with a higher case fatality rate (26%) as reported by other studies [4,5,14]. Underlying diseases could be a likely explanation, why patients with a lung focus have a poorer outcome than patient with an otogenic focus (36% vs. 14%, respectively, with Bonferonis correction: *P *= 0.19). However, an otogenic focus remained an independent prognostic factor after inclusion of other prognostic factors in the multivariate analysis.

Whilst several studies have investigated prognostic factors in pneumococcal meningitis, most of these studies have been smaller in size than the present study or have not applied multivariate statistics in their risk factor analysis [3-5,9-17]. The identification of other independent risk factors for fatal outcome than the focus of the infection in the multivariate analysis (convulsions) and in the univariate analysis (advanced age, having an underlying disease, presence of a lung focus, having a CT-scan prior to lumbar puncture, need for assisted ventilation, alterations in various CSF cytochemical parameters (e.g. low WBC counts, high protein and glucose levels)) confirms in part findings inconsistently obtained in previous studies [3-6,9-17].

Almost all patients with pneumococcal meningitis had fever and an altered mental status on admission (~94%), whereas nucal rigidity, the "classic sign" of meningitis, was present in 57% of patients. Consequently, some patients were admitted to the hospital suspected of having other kind of intracranial disease (e.g. apoplexy) than meningitis, and a diagnostic CT-scan was performed before the lumbar puncture with a subsequent delay in establishing the meningitis diagnosis and initiation of antibiotic therapy. Others have previously shown that a delay in the initiation of antibiotic therapy worsen the clinical outcome of community-acquired bacterial meningitis [22]. Thus, the results support international recommendation that patients presenting with fever and altered mental status should promptly have a diagnostic lumbar puncture or be given empirical antibiotic therapy and corticosteroids, and be sampled for blood culture before the CT-scan [23].

Less than 10% of patients were infected with pneumococcal strains with reduced susceptibility to penicillin, which was not related to a poorer outcome. Similar results have previously been found in countries with low or high levels of penicillin resistance [6,12,24]. Previously, we have shown that serotype-related differences (between serotype 1, 3, and 9 V) in mortality of pneumococcal meningitis exists [25]. The results from the present study may indicate that a relationship to mortality may exist for other serotypes, however, this has to be addressed in a larger patient population. The serotype coverage rate by the 7-, 9-, and 11-valent pneumococcal conjugate vaccine were 67%, 72%, and 86%, respectively, among children less than 2 years of age, and by the 23-valent vaccine it was 92% among adults, which is in accordance with previous published results obtained in Denmark during the period 1995–1999 [26]. No reliable data could be extracted from the present study on the vaccination status of the individual patients against pneumococcal infection due to incomplete data registration, but a more frequent use of pneumococcal vaccination may likely reduce the incidence of pneumococcal meningitis in Denmark, since vaccination of children and adults has reduced the risk for developing invasive pneumococcal disease in other countries [27,28].

The causes of death in patients with pneumococcal meningitis are multifactoral and involve both neurological and systemic complications [5]. Three patients died due to other causes (two patients due to gastrointestinal bleeding and one patient, because active therapy was stopped due of incurable cancer). Interestingly, no significant differences were observed in the causes of death between the five different groups of infection foci. Two out of every three patients died within the first week after admission, however, death directly related to pneumococcal meningitis still occurred up to 96 days after admission. Others have previously found that a mortality at 14 days as the endpoint is optimal to use when studying community-acquired bacterial meningitis [18]. Our results here and in a previous study suggest a longer study period, when studying case fatality in pneumococcal meningitis [25].

The frequency of sequelae due to pneumococcal meningitis was 41% with hearing loss in 26% of survivors and neurological sequelae in 16% of survivors, confirming previous results [1,4,6,10]. Sequelae occurred more frequently in adults than in children, most likely because pneumococcal meningitis is a less severe disease in children than in adults, as shown in the present study, or due to a higher susceptibility for the host to develop sequelae with increasing age. On the other hand, sequelae were detected more frequently in otogenic cases than in non-otogenic cases, despite a lower mortality rate in otogenic cases. However, there is one major bias in the evaluation of sequelae among meningitis patients, in particular with the evaluation of hearing loss, since it is not possible to compare the outcome with baseline parameters obtained before the onset of meningitis, which may have a significant impact on the interpretation of the results.

Whereas a beneficial effect of adjunctive therapy with corticosteroids on mortality did not reach statistical significance, surviving patients, who were treated with corticosteroids, had a significantly lower risk of developing sequelae than patients, who were not treated with corticosteroids. International guidelines and results from well-designed randomised trials have recommended the use of corticosteroids as adjunctive therapy of pneumococcal meningitis, when administered before or together with the first dose of antibiotics [1,23,29], but information's on the exact timing of corticosteroid administration could not be extracted from the medical records in the present study. Moreover, the majority of patients, who received corticosteroids in the present study, were children, because the study period was before the publication of the results of the European Dexamethasone in Adulthood Bacterial Meningitis Study in 2002 [1] and before the recommendation in Denmark to use corticosteroids as adjunctive therapy in adult bacterial meningitis [30]. Indeed, this affected the results significantly, and no beneficial effect of corticosteroid therapy was observed, when sub-analysis among children or adult cases were performed (*P *> 0.05, data not shown). However, the case fatality rate of pneumococcal meningitis will be expected to be lower in future studies performed after the implementation of corticosteroids as adjunctive therapy to all cases of bacterial meningitis.

Although new knowledge in the pathogenesis of pneumococcal meningitis has been obtained experimentally [31], the exact infection route into the CNS is not fully determined. Pneumococci may spread directly from an adjacent focus or via the haematogenously route into the CNS. In the present study, bacteraemia was present in 2 out of every 3 cases, and no significant difference in frequency of bacteraemia was observed between the 5 different groups of infection foci, indicating that pneumococci predominantly may spread haematougenously. However, bacteraemia also occurs secondary to meningitis, as shown in animal models after intracisternal inoculation [25]. Therefore, clinical studies with quantitative CSF and blood cultures from cases with different foci of the infection are required to provide further insight in the pathogenesis of pneumococcal meningitis.

The present study has limitations because not all data was collected prospectively, and several variables were unavailable from a number of cases leading to a decreased statistical power of the multivariate analysis. Also, we could not detect an association between degree of consciousness and case fatality rate, most likely because Glasgow Coma Scale tests were not performed on the patients, and thereby missing one of the most important predictors of a poor clinical outcome [5,6]. The results of the present study should be confirmed in future studies (i.e. in subanalysis of pneumococcal cases from prospective studies with less missing values [6]).

## Conclusion

Our results emphasize the prognostic importance of early recognition of a primary infection focus in pneumococcal meningitis. Meningitis should also be considered in patients presenting without nucal rigidity but with fever and altered mental status. Finally, a diagnostic lumbar puncture or start of antibiotic therapy and blood culture sampling should not be delayed by a CT-scan.

## Competing interests

The author(s) declare that they have no competing interests.

## Contribution of authors

CØ provided the scientific idea of the present study, designed the study, collected patient data, made the statistical analysis, and drafted the manuscript. HBK provided serotype data and participated in the design of the study. SS provided patient data from the notification reports and participated in the design of the study. All authors approved the final manuscript.

## Pre-publication history

The pre-publication history for this paper can be accessed here:


